# Diagnosis of Neonatal Sepsis: The Role of Inflammatory Markers

**DOI:** 10.3389/fped.2022.840288

**Published:** 2022-03-08

**Authors:** Julia Eichberger, Elisabeth Resch, Bernhard Resch

**Affiliations:** ^1^Research Unit for Neonatal Infectious Diseases and Epidemiology, Medical University of Graz, Graz, Austria; ^2^Division of Neonatology, Department of Pediatrics and Adolescent Medicine, Medical University of Graz, Graz, Austria

**Keywords:** early onset sepsis, late onset sepsis, preterm/full term infants, inflammatory marker, interleukin-6, C-reactive protein (CRP)

## Abstract

This is a narrative review on the role of biomarkers in the diagnosis of neonatal sepsis. We describe the difficulties to obtain standardized definitions in neonatal sepsis and discuss the limitations of published evidence of cut-off values and their sensitivities and specificities. Maternal risk factors influence the results of inflammatory markers as do gestational age, the time of sampling, the use of either cord blood or neonatal peripheral blood, and some non-infectious causes. Current evidence suggests that the use of promising diagnostic markers such as CD11b, CD64, IL-6, IL-8, PCT, and CRP, either alone or in combination, might enable clinicians discontinuing antibiotics confidently within 24–48 h. However, none of the current diagnostic markers is sensitive and specific enough to support the decision of withholding antibiotic treatment without considering clinical findings. It therefore seems to be justified that antibiotics are often initiated in ill term and especially preterm infants. Early markers like IL-6 and later markers like CRP are helpful in the diagnosis of neonatal sepsis considering the clinical aspect of the neonate, the gestational age, maternal risk factors and the time (age of the neonate regarding early-onset sepsis) of blood sampling.

## Introduction

Neonatal sepsis is still one of the leading causes of morbidity and mortality in the neonatal intensive care unit (NICU) (1). The symptoms are variable and non-specific (2). Diagnosis and treatment of neonatal sepsis remain challenging tasks even today (3). Early and efficient treatment is crucial for outcome and prognosis in neonatal sepsis cases, leading to frequent administration of empirically selected broad-spectrum antibiotics in high-risk infants (4, 5). Empirical treatment, however, increases the exposure to adverse drug effects, nosocomial complications and contributes to the development of resistant strains (6). In a cross-sectional study including 326,845 live births from 121 California hospitals with a NICU the percent of newborns with antibiotic exposure varied from 1.6 to 42.5% (7). Across hospitals, 11.4–335.7 infants received antibiotics per proven early-onset sepsis case and 2–164 infants per proven late-onset sepsis case (7). Withholding or delaying treatment in a potentially infected child, however, would be inacceptable given the rapid course and high fatality associated with neonatal sepsis (8). Biological markers that react rapidly after the onset of the inflammatory process are highly needed in the diagnosis of neonatal sepsis (9).

Neonatal sepsis is defined as either early onset sepsis (EOS) or late onset sepsis (LOS) based on whether onset of sepsis occurred before or after a certain neonatal age; and different ages have been used in the literature. According to the majority of studies EOS is defined as sepsis occurring within the first 72 h of life, with LOS presenting after this time period. This definition is also consistent with the EOS definition by the National Institute of Child Health and Human Development and Vermont Oxford Network (10). Early studies (11), but also more recent ones (12–14) on diagnostic accuracy of IL-6, e.g., were conducted in a study population of neonatal sepsis cases without further differentiation. However, it is important to distinguish between EOS and LOS, as inclusion of patients with different neonatal ages introduces a bias due to age-related risk factors (11). The definition of abnormal biomarker values and appropriate cut-offs might significantly depend on postnatal age (11).

A large cohort study with over 108,000 very low birth weight (VLBW) infants revealed a higher mortality rate of 25.9% in case of positive blood culture compared to 11.3% for infants with negative cultures, and higher rates for EOS than for LOS (15.1% compared to 8.5%) (15). In term neonates much lower mortalities were found. In a cohort of more than 140,000 term infants mortality was 0.8% in infants with EOS vs. 0.2% in those without (16).

### Early vs. Late Onset Sepsis

Neonates have only a limited repertoire of stereotypic reactions to different harmful stimuli either infectious, metabolic, respiratory, or traumatic (17) and many symptoms or signs of sepsis can be attributed to non-infectious neonatal disorders (18). EOS, in particular, presents with a different clinical course and involves other pathogens than sepsis later in life (19). Its great morbidity, mortality, and lack of early and reliable diagnostic tools make the management of EOS so challenging for the clinician (20). The incidence of culture-proven EOS in the United States is estimated to be 0.77–1 per 1,000 live births, however for infants with a body weight of <1,000 g, higher incidences of 26 per 1,000 are estimated (21).

EOS is mostly caused by transmission of microorganisms from the mother, happening either prenatally due to ascending colonization following the rupture of membranes or perinatal during the passage through an infected birth canal (5). Hence, causative pathogens are typically found in the maternal vaginal and fecal flora (22). Less common but also possible is an infection via the haematogenous route (19). Microorganisms prevalent in the labor or delivery room may also cause infection of the neonate (23). Simonsen et al. (21) reported Group B streptococcus (GBS) as the most common causative organism. Intra-partum antimicrobial prophylaxis however has led to a significant reduction of GBS infection rates (24). E. coli is the microorganism with the highest mortality in EOS (21).

Infants at the NICU are highly susceptible to LOS (25). A multicenter survey by Stoll et al. (26) suggested that 21% of VLBW infants who survived >72 h of age had at least one episode of septicaemia. In extremely low birth weight (ELBW) infants nearly two thirds experience more than one episode of suspected or culture-proven LOS during hospitalization (27). LOS has been associated with poor neurodevelopment and growth and with altered lung development (28). The most common causative organisms for nosocomial infections in neonates admitted to NICUs are Gram-positive cocci, especially coagulase-negative staphylococci (29). IL-6 has been shown to be superior to CRP in the diagnosis of late-onset neonatal sepsis due to coagulase-negative staphylococci (29). Its combination with CRP adds important information regarding withholding or stopping antibiotic therapy (29). Strunk et al. (30) hypothesized that a perinatal inflammation process might support the functional maturation of the preterm immune system, thus providing protection against LOS.

### Preterm vs. Term Neonate

Consideration of gestational age in the septic infant is important for a variety of reasons. The neonatal immune system may not be fully developed (31) and cut-off values of diagnostic markers might depend on gestational age (32–35). Delayed maturation of the specific humoral and cellular immune response of neonatal B and T cells, defective activation of the complement system, and deficiencies of the myelopoetic system in the neonate have been discussed (19). Although a defective cytokine production of neonatal cells has been observed *in vitro, in vivo* studies did not uniformly confirm these findings (19). It has further been hypothesized that preterm infants might have a completely different immune response to sepsis than those born at term (20). Berner et al. (19) however, found that the predictive value of cord blood cytokine levels for the development of EOS does not depend on maturity and holds true for preterm infants. Yoon et al. (36) stated that the preterm fetus upon microbial invasion of the amniotic cavity is capable of mounting a systemic cytokine response, quantifiable by peripheral blood IL-6 levels. Prophylactic antibiotic treatment is often given to neonates with gestational age below 32 weeks to account for the presumed increased susceptibility to infections due to immunologic immaturity (4).

While signs of sepsis are generally subtle and unspecific in the neonate, sepsis presentation is often even more subtle in the preterm infant (3). On the other hand, preterm infants are more likely to present clinical signs like respiratory distress, apnoea, bradycardia, temperature instability, and cyanosis (3). While they could indicate sepsis, they might as well be a result of respiratory distress syndrome or prematurity itself (3, 37).

Preterm infants are more frequently born in the context of intrauterine infection (3). Infection itself has the potential to induce (preterm) labor via the secretion of pro-inflammatory cytokines secreted by the mother and/or the fetus (in response to infection) (38). Preterm infants are more likely to present with symptoms at delivery, while the majority of term neonates with EOS develops symptoms after delivery (31). These findings indicate that for preterm infants exposure to bacteria is more likely to happen *in utero*, while term neonates might be exposed to bacteria later, possibly during the passage of the birth canal (31).

Preterm labor with intact membranes and preterm premature rupture of the membranes (pPROM) are conditions frequently associated with intra-amniotic infection and inflammation (38). Neonates with pPROM have an increased risk (4–33%) of infection (39, 40). The relationship between pPROM, fetal inflammatory response syndrome (FIRS), and neonatal sepsis in preterm infants has been subject to research (39). Romero et al. showed that in patients with pPROM, presence of FIRS led to the onset of preterm parturition (41). It is therefore not surprising that neonates with FIRS were found to have lower gestational age and lower birthweight than neonates without (22).

Morbidity and mortality of infection is particularly high in infants delivered prematurely, either due to preterm labor or preterm premature rupture of the membranes (8). The percentage of fatal neonatal infections is higher the lower the gestational age is (42) and the risk of death is 120-fold higher in preterm born population than in those delivered at term (3). Even though cord blood IL-6 levels and presence of funisitis have been found to be independent predictors of neonatal morbidity, prematurity is still considered the leading cause of perinatal morbidity and mortality (3, 6).

## Diagnosis of Neonatal Sepsis

A positive microbiological blood culture poses the gold standard for the diagnosis of neonatal sepsis. However, much controversy exist as to the correct blood volume in neonates (43). Especially in cases of low level bacteraemia, which may account for up to two-thirds of neonatal sepsis cases, larger volumes than feasible might be needed (44). For blood samples, seeded with common neonatal pathogens, Schelonka et al. (45) demonstrated, that the sensitivity of blood cultures approaches 100% for 1 mL of inoculated blood with a bacteremia of at least 4 colony-forming units (CFU) per milliliter. Maternal antibiotic therapy under birth, while important in the prevention of neonatal sepsis, has further been discussed as possible confounder of blood culture results (46). The knowledge of these limiting factors in the group of neonates, together with high numbers of negative blood cultures in neonates with risk factors or clinical signs of EOS have led to discussions regarding the sensitivity of blood cultures in neonates (46). The reason for the high number of culture-negative cases is not clear, and diagnostic criteria used in the different publications vary substantially, so that an alternative explanation might be over-diagnosis of sepsis among non-infected infants (46). So, while sensitivity of blood culture in neonates is often quoted to be low, Cantey et al. (47) argue that there should be more focus on correctly drawn blood cultures and consequently trust in negative culture results. With results being available only after 2–3 days, time to diagnosis would be unacceptable high and hamper the use of blood cultures for early detection of sepsis (5). A recent retrospective observational study, however, showed that of 40 positive blood cultures, collected from late preterm and term infants, 39 (98%) were showing bacterial growth within 24 h. The possibility of cross-contamination or asymptomatic bacteremia might also result in a misleading or inaccurate diagnosis (44, 48).

None of the widespread laboratory markers of infection [C reactive protein (CRP), white blood cell count (WBC), absolute neutrophil count (ANC), immature to total neutrophil ratio (IT-ratio)] has enough sensitivity or specificity to detect all infected children (17, 49). Their diagnostic value might be especially limited in the early course of the disease (49). CRP, for example, is known to rise not earlier than 12–24 h after the onset of neonatal infection (2). In addition, Leucocyte count, IT- ratio, and ANC could not distinguish infected from control infants in an early sepsis evaluation (9). Hematologic tests perform poorly in differentiating between sepsis and non-infectious conditions (50). Thrombocytopenia although suggestive of systemic infection is also seen in severe lung disease due to platelet sequestration and thus not specific enough (50). The IT-ratio is determined by identifying immature neutrophil forms on a peripheral blood. Hence, its value as a diagnostic marker depends on skilful technicians. We published data on a significantly increased number of either immature granulocytes or immature myeloid information in neonates with EOS compared to controls and found their automated determination to be a useful adjunctive method in the diagnosis of EOS (51). Serial measurements of lymphocyte subsets [CD3+, CD4+, CD8+, natural killer (NK) cells, and B cells] in preterm neonates with late-onset sepsis and infection-free controls showed higher percentages of NK and B cells in the sepsis group, while those of CD3+, CD4+, and CD8+ showed no differences (52). Clinical management, especially decisions for antibiotic treatment, can't be based solely on hematologic markers (50).

Benitz et al. (44) concluded that the best-established use for laboratory markers, including hematological markers, acute-phase reactants, and inflammatory cytokines, lies in the retrospective determination that an infant was not infected, based on failure to mount an acute-phase response over the following 24–48 h. In that case, the use of these markers would offer no improvement compared to blood culture.

16S rDNA is a DNA region common to all bacteria, its detection via 16S rDNA PCR has been discussed as an alternative or addition to blood culture (24, 53, 54). PCR is not only faster it is also already considered as the gold standard in the detection of neonatal meningitis caused by herpes simplex virus (54). Al-Zahrani et al. (24) found a higher sensitivity (39 vs. 35.2%), but a lower specificity (80.5 vs. 93.5%) in comparison to blood culture for the detection of EOS in a group of proven (positive blood culture and/or positive PCR results) and clinical sepsis cases. In this study PCR was positive in 23 out of 25 blood culture positive cases (24), in another study however only 7 out of 17 cases were detected (54). Again, standardized and clinical evaluated assays for bacterial DNA detection in neonatal blood samples are lacking (24).

The options for antepartum detection of high risk fetuses are limited to historical factors, maternal clinical status, fetal behavioral assessment and the detection of amnionitis via amniocentesis (8). Risk factors for neonatal sepsis include prolonged rupture of membranes, chorioamnionitis, colonization with Group B streptococcus, prematurity, perinatal asphyxia, male gender of the fetus, foul smelling amniotic fluid, and urinary tract infection (8). However, no risk factor has consistently been able to identify a significant portion of infants with neonatal sepsis (8).

Therefore, sepsis diagnosis in neonates is typically based on a combination of maternal risk factors, hematologic indices and the judgement of the physician rather than their clinical presentation (3, 11). Early biomarkers combined with accurate and rapid measurement methods are urgently needed for early diagnosis of sepsis and guidance of antibiotic therapy (55).

The sepsis calculator https://neonatalsepsiscalculator.kaiserpermanente.org/, a tool developed by Kaiser Permanente, provides recommendations for clinical management ranging from routine care to administration of empiric antibiotics and has been found useful for decreasing empiric antibiotic use in suspected EOS (56). A recent meta-analysis including 18 studies, with over 459,000 newborns, however, found that at initial evaluation its application assigns frequent vital signs or routine care to a substantial proportion of EOS cases, 15 and 44%, respectively (57). By 12 h of age these numbers decreased to 11.1%, and 27.8%, respectively. It is therefore important to note that the use of the EOS Calculator involves clinical monitoring beyond the initial risk classification, and clinical vigilance remains essential for all newborns (57). Since it was designed for late preterm and term neonates, the EOS calculator does not cover the high-risk population of vulnerable preterm.

Newer biomarkers investigated include acute phase proteins, cytokines and cell surface antigens (20). As mediators of the inflammatory cascade, elevated levels are likely to be observed as response to infective as well as non-infective inflammatory triggers, such as toxic processes and tissue damage (58). Ventilation may cause an inflammatory reaction in the lungs, and inflammatory markers leaving the alveolar space might appear in the systemic circulation [63]. Small sample sizes, inconsistent definitions of sepsis, heterogeneity of the study population and differences between cut-off values led to inconclusive results (20). A study (19) comparing the mRNA expression of various inflammatory markers (G-CSF, TNF, IL-1β, IL-6, and IL-8) in umbilical cord blood cells to their plasma levels in the same blood sample, found that, with the exception of TNF, mRNA expression in septic infants was not more frequently detectable than in non-septic ones. Cord blood plasma levels but not the presence of mRNA could predict EOS. Absence of mRNA could indicate that maternal cells are the origin of the cytokine production, with cytokines reaching the fetal circulation via placental transfer (59). Berner et al. (19) compared neonatal cytokine levels to the corresponding maternal blood levels. Cord blood levels of G-CSF, IL-1β, IL-6, and IL-8 were significantly higher in septic infants than in their mothers. The authors (19) therefore hypothesized that the cytokine production was triggered by an infection that occurs before birth around the time of delivery. In that case, mRNA levels might have already decreased to rarely detectable levels at the time of birth. Additionally, cell types other than mononuclear blood cells, i.e., umbilical endothelial cells, might be involved in the cytokine secretion. Production of cytokines in the gastrointestinal tract as response to ingestion of infected amiotic fluid has been discussed as a potential source of cytokines in infants with clinical sepsis syndrome and negative blood culture (3).

### Systemic/Fetal Inflammatory Response Syndrome (SIRS/FIRS)

Clinical manifestations of sepsis are not limited to patients with infections, they can also be observed in patients suffering from, e.g., burns, trauma, pancreatitis, ischemia, or immune-mediated injury and result from a systemic inflammatory process (60). This phenomenon was termed “Systemic Inflammatory Response Syndrome” (SIRS) and was diagnosed if two or more of the following criteria are met in adults: Temperature >38°C or <36°C, heart rate >90 beats/min, respiratory rate >20 breaths/min or PaCO_2_ <32 mmHg, white blood cell (WBC) count >12,000/mm^3^ or <4,000/mm^3^ or >10% immature bands. In the neonatal field heart rate should be >180/min and tachypnea > 60/min, WBC below 6,000/mm^3^ or above 30,000/mm^3^, and immature to total neutrophil ratio >0.2. Since vital signs, with exception of the fetal heart rate, and white blood cell counts cannot be readily determined before birth, the definition of SIRS cannot be applied to the human fetus (38).

Presence of fetal systemic inflammation akin to that observed in adult patients with SIRS was termed “Fetal Inflammatory Response Syndrome” (FIRS) and defined as an elevated concentration of fetal plasma interleukin-6 >11 pg/mL (61). Other cytokines like TNF and IL-1β were not always detected in peripheral blood with the assays available at the time (38). Its role as a major mediator of the acute phase response to infection or tissue injury further justified the choice of IL-6 as the marker of inflammation (38). Interestingly the cut-off value of 11 pg/mL obtained with ROC analysis, coincided with the two standard deviations above the mean IL-6 value in a population of 29 fetuses with subsequent normal pregnancy outcome (61). The authors found that FIRS was an independent risk factor for the occurrence of severe neonatal morbidity (61). The histopathologic counterpart of FIRS is the presence of funisitis, a polymorphonuclear leukocyte infiltration along the umbilical cord in response to infection (6, 38). Funisitis is considered to be the last stage of intra-uterine infection and, like elevated IL-6 levels, is associated with a worse neonatal outcome, including the risk of EOS (6). Despite the immaturity of the innate immune system, transcriptome analysis of patients with FIRS showed remarkable similarities between FIRS and its adult counterpart SIRS (62).

Jung et al. (38) concluded that FIRS, with the extended definition of elevation of cytokines in umbilical cord blood, presence of acute phase reactants, or severe funisitis, in preterm neonates was a risk factor for early neonatal sepsis. We found a possible association between FIRS with an adverse neonatal outcome defined as hospital mortality and/or presence of any of five morbidities including early and late onset sepsis (22). Thus, we were able to demonstrate an association between FIRS and EOS, with higher cord blood IL-6 levels in neonates with culture proven and clinical EOS. The presence of LOS however, did not show increased IL-6 levels and could not be associated with FIRS (22). In the search of an appropriate cut-off value, it is of interest that the risk for an adverse neonatal outcome correlated with the magnitude of cord blood IL-6 (22). Presence of FIRS led to a 6-fold increase in risk, values >50 pg/mL to a 9-fold and values >500 pg/mL to a 30-fold increase for an adverse neonatal outcome (22). Similar to our results (22), Strunk et al. (30) evaluating the link between histological chorioamnionitis (HCA) and neonatal sepsis showed that HCA was associated with increased risk for EOS, but seemed to reduce the risk for LOS. The authors hypothesized that the perinatal inflammation process might enhance maturation of the neonatal immune system and therefore indirectly decreases an infant's risk for developing LOS. In contrast to these findings Jung et al. (38), pointing to the fact that LOS has been observed in infants born with FIRS after an initial period of clinical improvement, suggested that the anti-inflammatory response might play an important role in the development of LOS. Increased concentrations of anti-inflammatory cytokines like IL-10 measured in infected infants (42, 49) proof the activation of an anti-inflammatory immune response and support this theory (38).

### The Ideal Biomarker

Giving thought to the requirements of an ideal diagnostic marker for neonatal bacterial infection has to fulfill, Ng (58) proposed a set of clinical and laboratory criteria. The authors (63) later extended their criteria according to a demand for more clinical information. Additional items include prediction of disease severity and provide an algorithm for antimicrobial treatment.

Considering the mortality and morbidity of neonatal sepsis, which is particularly high in preterm and VLBW infants (15), it is more important for a diagnostic test to have the highest possible level of sensitivity than the highest level of specificity (9). Ng (58) suggested a sensitivity (infected infants have a positive test) and negative predictive value (a negative test confidently rules out infection) approaching 100%, as not to withhold or delay treatment due to false negative test results (58). Specificity (the test is negative in non-infected infants) and positive predictive value (a positive test indicates true infection) should be reasonable high, i.e., above 85%, to minimize the unnecessary use of antibiotics in false positive cases (58). Such a test would not only reduce the need for extensive neonatal evaluation and empiric antibiotic treatment, but also reduce the costs related to the care of the preterm infant (37).

Tests or biomarkers with a high turnaround time are only capable to guide the discontinuation of empirical antibiotic treatment upon negative test results—a common approach in NICUs (49). An ideal marker however would be able to guide the clinician on whether to start treatment at the onset of non-specific clinical signs (63). Identification of the causative microorganism and its antibiotic susceptibility would further allow for targeted antibiotic therapy (63). Prediction of disease severity and prognosis would help the clinician at identifying those infants who are most in highly need of urgent treatment and intensive care support (63). Keeping the sample size small, i.e., a blood sample <0.5 mL, is important in neonates, especially in the group of very low birth weight infants (49, 63).

The utility of a biomarker also depends on its ability to serve as a routine diagnostic test. Specimen collection depends on clinical working hours and might be performed at different time periods in regard to sepsis onset. Hence a biomarker should be biochemically stable and maintain up- or downregulated for at least 12–24 h (63). The biomarker concentration at testing should reflect the status of the infant at the time of specimen sampling, even after transportation and storage of the sample (63). To provide the rapid turnaround time required for the early identification and appropriate management of true sepsis cases (i.e., specimen collection reporting on results <6 h), automated essays or on-demand testing in clinical laboratories might be required (63).

The characteristics of the ideal biomarker according to Ng and Lam (63) are summarized in Table 1. Although the list was formulated in the context of LOS, its principles probably hold true for EOS diagnosis.

**Table 1:** Criteria for an ideal biomarker or test for the diagnosis of neonatal sepsis (63).

**Clinical properties**
1. Such biomarkers should have a well-defined cut-off value and a sensitivity and negative predictive value approaching 100% for “ruling out” LOS (but simultaneously having high specificity and positive predictive value >85%) 2. Detect infection early (i.e., at clinical presentation) 3. Identify a specific pathogen or a category of pathogens (e.g. viral, bacterial, and fungal organisms; gram-positive organisms vs. gram-negative organisms; a specific species of pathogen) 4. Monitor disease progress and guide antimicrobial treatment (e.g. bacterial antibiotic resistance gene detection) 5. Predict the disease severity at the onset of infection (e.g. identify the type of virulent pathogen, predict DIC at the onset of disease presentation) 6. Predict prognosis (i.e., mortality)
**Laboratory properties**
1. Stable compound that may allow an adequate time window for specimen collection within normal working hours (i.e., sustained increase or decrease in biomarker level for at least 24 h) or easy storage of the specimen without significant decomposition of the active compound until laboratory processing 2. Quantitative determination of biomarker concentration 3. Automatic and easy method of measurement 4. Quick turnaround time (i.e., specimen collection, transport, laboratory processing time, and reporting of results to clinicians within 6 h) 5. Small volume of specimen (i.e., <0.5 mL blood) 6. Daily or on-demand availability of testing in clinical laboratories 7. Low-cost test that can be used as a routine measurement

Once a sensitive and specific marker with a rapid reliable assay is found it must be subjected to large-scale evaluation (37). The cut-off value, i.e., the biomarker separating infected from non-infected children should be determined in a well-defined patient population using ROC analysis, thus allowing for comparison of results between NICUs (58). Most biomarker studies already rely on ROC analysis to define cut-off levels. However, within ROC analysis there are various methods to determine the ideal cut-off value. The three most common criteria for definition of a cut-off point are the following: (1) Selection of the point on the ROC curve where sensitivity and specificity are almost equal. (2) The Youden's index (sensitivity + specificity – 1) meaning the point resulting in the highest sum of sensitivity and specificity. (3) The point with the minimum distance to the upper left corner of the plot (64).

The area under the ROC curve (AUC) is an estimator of the overall accuracy of a diagnostic test (65). Biomarkers are commonly considered good or excellent if their AUCs are >0.75 or >0.9, respectively (66), the interval between 0.7 and 0.9 indicates moderate diagnostic accuracy (67). However, not all studies use these predefined definitions and moderate diagnostic accuracy was reported for AUCs as low as 0.65 (40) and high diagnostic accuracy for AUCs of 0.751 (48). Investigators should further be aware of the need to calibrate their assays using an international standard in order to compare results among laboratories and studies (38).

### Inflammatory Markers and Their Dynamics in Neonatal Sepsis

**Figure 1:**
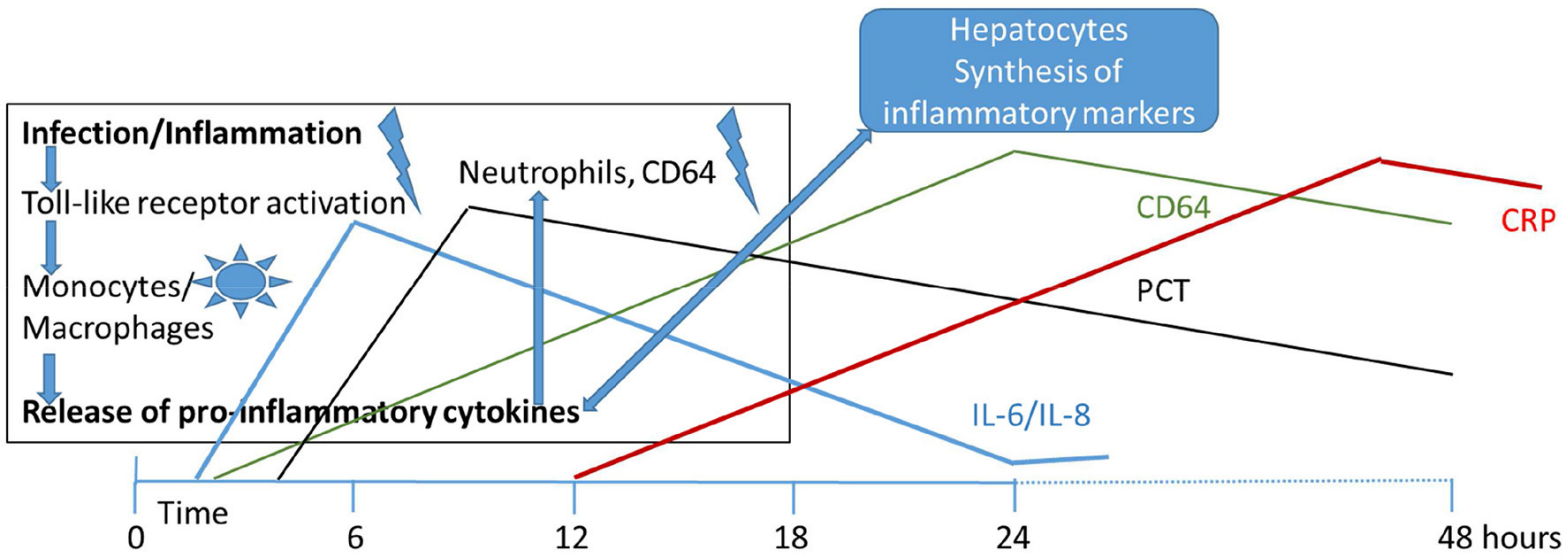
Initiation of infectious/inflammatory processes and release of inflammatory markers over the first 48 h of life in early onset neonatal sepsis. IL, interleukin; PCT, procalcitonin; CRP, C-reactive protein; CD64, cluster of differentiation (neutrophil surface expression).

Figure 1 illustrates the inflammatory cascade showing the involved cell types and biomarkers over time [adapted from (68)]. According to their appearance and disappearance in the course of disease, the markers have been classified into early, intermediate and late markers of sepsis. Figure 1 also shows how the level of these biomarker rise and fall during the first 48 h after onset of sepsis. Elevated in the early phase of infection are the interleukins IL-6 and−8, and CD64, ICAM, TNF, and IFN-γ, followed by the acute phase proteins PCT and CRP in the mid and late phase, respectively (69).

#### Cytokines and Chemokines

Upon pathogen induced activation of toll-like receptors (TLRs), proinflammatory cytokines are released from monocytes and macrophages during the early phase of the immune response starting the inflammation process (70). Cytokines have found to be promising markers of bacterial sepsis in the newborn infant (71). However, problems with invasiveness, response time, and specificity remain to be solved (72).

Tumor necrosis factor (TNF) is a proinflammatory cytokine of the first line immune response (19). It stimulates IL-6 production and acts on several types of target cells, both immune and non-immune (3). IL-6 has an inhibitory power on TNF, acting at the transcription level as well as through stimulation of the synthesis of TNF soluble receptor (3, 73). Inhibitory effects on TNF production have also been reported for prostaglandin E_2_ (3). TNF levels rise rapidly, with peak blood levels at ~1 h after the stimulus, and disappear from circulation within 3 h (9). In a study by Kashlan et al. elevated concentrations of IL-6 but not TNF were associated with infection (3). They hypothesized, that at the time of sample collection, the inflammatory response in cases of congenital sepsis had already progressed past the rapidly peaking TNF secretion (3). Leaving the resultant IL-6 elevation, as a sign that stimulation by TNF has already taken place (3).

The cytokine IL-6 is a particularly early marker of neonatal sepsis. It is released within 2 h after the onset of bacteraemia, peaks at ~6 h and finally declines over the following 24 h (68). IL-6 levels are significantly elevated up to 48 h prior to the onset of clinical sepsis (74). IL-6 is characterized by a short half-life due to binding to plasma proteins such as α2-macroglobulin, early storage in the liver or inhibition by other cytokines (75). While some investigators have found that the neonatal IL-6 response is comparable to that found in adults, others have reported a diminished IL-6 production (2, 76). Stress and tissue injury have the potential to provoke an IL-6 response (58, 73). Interpretation of IL-6 levels for diagnosis of neonatal sepsis therefore might be hampered by underlying illnesses and their severity. To improve the diagnostic capacity of this early marker, combinations with later and more specific biomarkers (e.g., CRP) have been suggested (77). A relatively large sample size is required since IL-6 circulates at rather low levels (17). Findings in healthy infants indicate a negative correlation between gestational age and IL-6 levels (35).

The cytokine Interleukin-8 (IL-8) plays a role in the release, activation and chemotaxis of neutrophils (14). Increased IL-8 serum levels have been reported in both early- and late- onset neonatal sepsis (14). In a meta-analysis including eight studies with a total of 548 neonates pooled sensitivity and specificity of IL-8 were 78 and 84%, respectively (78). Like IL-6, IL-8 has a short half-life and its diagnostic properties have been shown to increase upon combination with CRP (28).

#### Acute Phase Proteins

Hepatic synthesis of the acute phase protein CRP as a response to bacterial infection takes place after stimulation by IL-6 and other proinflammatory cytokines. After synthesis, CRP in turn leads to activation of the complement system, increased phagocytosis, activation of macrophages and monocytes, and elevated production of proinflammatory cytokines (79). CRP levels begin to rise between 10 and 12 h after onset of infection, and peak at 48 h (33, 79). A relatively long serum half-life of 24–48 h has been reported for CRP (33). Due to the delayed rise of CRP levels as a response to infection CRP has an unacceptable low sensitivity within the first 24 h, i.e., for the early diagnosis of neonatal sepsis (33, 80). It further shows a non-specific physiological increase in the first 3 days of life, probably due to the stress of delivery and other non-infectious perinatal and maternal factors, hampering its use as a sepsis marker during this time period (81). Levels of up to 20 mg/L at 48 h after delivery have been reported in healthy neonates (82). Performance of serial measurements and combination of CRP with earlier markers such as CD64, interleukins or procalcitonin have the potential to improve the diagnostic accuracy in the early phases of sepsis in both EOS and LOS (33, 81). Beyond its use as diagnostic marker, CRP is particularly useful in monitoring the response to treatment and is used to guide the antibiotic therapy in neonatal sepsis (33). Benitz et al. (44) demonstrated that a persistence of normal CRP levels under antibiotic treatment strongly correlates with the absence of infection. Serial measurements are the most accurate and reliable in the diagnosis of bacterial infection of the neonate and are recommended within a time window of 24–48 h after onset of infection (21, 33, 83). Established by Mathers and Pohlandt (83), the most commonly used cut-off value for CRP during the first days of life continues to be 10 mg/L (33). By use of ROC analysis we demonstrated that CRP could play a role in the early diagnosis of neonatal sepsis if cut-off values were lowered (80). Perrone et al. (82) performing a study on CRP levels in healthy neonates stated that a static cut-off level is unable to reflect the physiological kinetics of CRP, and proposed the use of different cut-off levels adapted to gestational age, postnatal age and mode of delivery. This was confirmed by our group (32, 33) demonstrating that preterm infants had lower CRP values compared to term infants. CRP values increased by 0.405 mg/L for every 1 week increase in gestational age (32, 33). Raised CRP levels are not specific for bacterial infection, and might also appear in conditions as asphyxia, shock, intraventricular hemorrhage, surgery, and meconium aspiration (84). For viral infections only slight elevations of CRP levels (<5 mg/L) have been reported (85, 86). Non-infectious inflammatory processes, such as PROM, meconium-stained amniotic fluid and prolonged labor, however, also caused significant elevations of CRP (82). Advantages of CRP as sepsis marker include its broad availability, simplicity, speed, and low cost (82). CRP refers to high sensitive CRP (hsCRP) when high-sensitivity assay techniques are used to measure concentrations as low as 0.01 mg/L (87).

Procalcitonin (PCT), the prohormone of calcitonin, is mainly produced by monocytes and hepatocytes and shows a significant elevation during infections in neonates, children and adults (88). Elevated PCT levels as a response to infection can be detected within 6 h after its onset, peak at 18–24 h and remain elevated up to 48 h (the half-life of PCT in peripheral blood is ~24 h) (88). Hence, PCT classifies as an early to intermediate-rising biomarker. PCT, like CRP, shows a physiological increase after birth, limiting its diagnostic value in the first 2–4 days of life (81). Studies evaluating the potential of PCT as an early marker for neonatal sepsis (89–91) have found that within the first 48 h of life elevated PCT levels were present even in uninfected or healthy neonates. However, the rise in PCT levels is much more marked in bacterial and fungal, but not viral, infections (92). Reference values and age related 95th percentile nomograms for the first days of life exist for healthy term and preterm infants (89, 92, 93) and have served as basis for the use of age-adjusted cut-off values (5, 94). In children and neonates after 72 h of age, PCT values <0.5 ng/ml seem to be normal; increases to 0.5–2 ng/ml seem to be related to non-infectious inflammation, viral or focal bacterial infections and increases above 2–2.5 ng/ml, seem to be related to bacterial or fungal systemic infections (95). In a recent multicentre, randomized controlled trial (NeoPIns) Stocker et al. (96) evaluated the potential of PCT to guide antibiotic treatment in infants with suspected EOS. They found that, within a population with low incidence of culture-proven infection, discontinuation of treatment based on PCT resulted in no adverse outcomes and duration of antibiotic therapy was significantly reduced (96). In a meta-analysis assessing the diagnostic potential of PCT in neonatal sepsis, the diagnostic accuracy seemed to be higher in neonates with LOS, than in those with EOS (5, 97). However, fewer data were available for LOS. Statistical heterogeneity and differences in the definitions used for neonatal sepsis additionally had to be taken into account. Advantages of PCT include its wide diagnostic window (88), its specificity to bacterial infections (98) and its quick reduction in response to adequate therapy (99). PCT also proved to play a role in SIRS and increased levels of PCT were associated with increased severity of disease and increased rates of mortality in adults (100).

Synthesis of the acute phase protein Serum amyloid A (SAA) is regulated by the pro-inflammatory cytokines IL-6 and TNF and takes place mainly in the liver, but also happens in smooth muscle cells, macrophages, adipocytes, and endothelial cells (81). The effect of SAA is mainly anti-inflammatory. It reduces the production of prostaglandin E_2_ and the oxidative respiration of neutrophils, counteracts the pyrogenic effect of a number of cytokines, inhibits platelet activation, negatively controls the production of antibodies, and induces the secretion of collagenase by fibroblasts (101). Free SAA has been found to possess cytokine-like properties which induce chemotaxis of neutrophils, granulocytes, and T-lymphocytes (102). Differences to the acute phase protein CRP include an earlier and sharper rise in the acute phase response, which occurs not only in bacterial but also in viral infections (101). A study set out to define normal SAA levels in healthy individuals and reported median SAA levels of 0.758 mg/L (range: 0.758–3.000) for cord blood and 1.516 (0.758–10.580) for 35 neonates in each group (103). During neonatal sepsis a 1,000-fold increase in the serum concentration of SAA has been reported (104), and elevated levels have been found in early- as well as in late onset sepsis (105–107). In a meta-analysis, consisting of a total of nine studies, diagnostic accuracy of SAA in EOS and LOS (measured 8–96 h after onset of infection) was found to be moderate and comparable to those of CRP and PCT (108). However, findings were again limited by heterogeneity of study population groups and cut-off levels. Other studies reported improved sensitivity relative to CRP at the point of clinical suspicion (106, 107). An advantage of SAA is the availability of an automated and rapid test, however the number of studies about the SAA test in neonatal sepsis is limited (108, 109).

#### Cell Surface Molecules

The Fcγ receptor 1 alpha chain, known as Cluster of differentiation 64 (CD64) is an immunoglobulin binding receptor found on the surface of leukocytes and showing high affinity to IgG immunoglobulin (110). In bacterial infection stimulation by proinflammatory cytokines IFN-γ and TNF and granulocyte colony stimulating factor (G-CSF) leads to an upregulation of CD64 expression (5–10-fold in comparison with baseline levels) (111, 112). The increase of CD64 is associated with the intensity of the triggering cytokine release (113). CD64 in turn induced enhanced antigen presentation, and facilitated phagocytosis and intracellular killing of opsonized microbes (110, 114).

In healthy subjects, antigen-presenting cells (monocytes, macrophages, and dendritic cells) express CD64, while neutrophils show only very low levels of CD64 expression (115). The later however rises by ongoing infection, converting it into an interesting sepsis marker. The neutrophil CD64 expression, often referred to as nCD64, is measured either alone or as a ratio to the monocyte CD64, which did not increase significantly (116). During bacterial infection nCD64 expression was markedly increased in all age groups, but, interestingly, higher levels were found in healthy preterm and term neonates when compared with healthy adults (115). Increased nCD64 expression was detected within 1–6 h after bacterial invasion and levels remained elevated for >24 h while viral infections were not associated with an increased nCD64 expression (112). Promising results were published for CD64 as a diagnostic marker in both EOS and LOS, but study heterogeneity led to a wide range of sensitivity and specificity, respectively (81). Increased CD64 expression has been considered as an independent risk factor for LOS, which has to be taken into account when its diagnostic value is evaluated in LOS (117). Advantages of CD64 as sepsis marker include the wide diagnostic window, the very small amount of blood needed (±50 μL of whole blood), easy handling and rapid turnaround time being <1 h (81, 118, 119). Serial measurements of CD64 were suggested for guiding antibiotic therapy in neonates (120). CD64 quantitative flow cytometric analysis could be developed into a routine clinical test with high comparability and reproducibility across different laboratories (121). However, to this date there is a lack of consistent cut-off values for CD64 and further research is needed to define the optimal cut-off value and time point of measurement, before CD64 expression testing could be incorporated in the clinical practice (122, 123).

## Discussion

Despite the promising results reported by many studies, most diagnostic markers fail to meet the criteria required for clinical practice. Cost, availability of specimens at the appropriate time, complexity of the assay methods, laboratory turnover time, reliability of the tests, and experience of the attending clinicians are all important factors in determining the accuracy of a diagnostic marker for clinical use (58).

Assays of chemokines and cytokines, as well as tests measuring the expression of cell surface antigens are expensive (58). However, 10% of all deliveries are preterm births and most of them have complete blood cell counts and intravenous access. If further sepsis evaluation is added, blood cultures (costs at $65 per set) and lumbar punctures (costs at $162 per procedure, $129 for cultures, cell count, protein, and glucose studies) add to an economic impact of current infant sepsis evaluation that is impressive (43). While the costs for 3 days of empiric antibiotic therapy with ampicillin and gentamicin are moderate between $12 and $15, high facility costs are generated due to prolonged NICU stays (37). Hence, effective testing strategies that enable a reduction of extended sepsis evaluation and empirical treatment would result in tremendous cost savings (37).

The usefulness of new inflammatory markers depends on the laboratory turnover time. Performing analyses in batches, or if possible (CD64) postponing sample collection until the next working morning, hampers their use as “early warning markers” (58). Finally, a more recent meta-analysis revealed low sensitivities and specificities and, thus, concluded to use it cautiously in the diagnosis of neonatal sepsis (also poorer performance in preterm than tem infants) (124). To satisfy the rapid turnover time required clinically a trained technician needs to be available at all times, something not practicable in most institutions (58). Therefore, *ad-hoc* measurement will only become cost effective if assay methods become automated (58).

As with blood culture the limiting factor for measuring combinations of cytokines is the large volume of serum required. Conventional enzyme-linked immunosorbent assay techniques require about 100–250 μL to quantify one protein (49). Something not feasible, especially in very low birth weight (VLBW) infants (49). Multiplex systems based on flow cytometry allow for the simultaneous quantitative measurement of several biomarkers with only a minimal volume of blood (49). For example only 50 μL of plasma are required for the measurement of six cytokines, or 50 μL of whole blood for each surface antigen measurement (49, 58). However, these are not the typical platforms used to quantitate analyses in clinical medicine (38) and intention-to-treat studies are required to examine their potential for reducing unnecessary antibiotic treatments (49). Ng (58) saw the use of the flow cytometric technology in the identification of cytokines or cell surface markers most suitable for clinical use.

A recent analysis (125) of 480 episodes of suspected LOS in 208 preterm infants below 32 weeks of gestational age showed that serum IL-6 and PCT levels (hazard ratios 2.28 and 2.91, respectively), but not CRP (hazard ratio 1.16), were associated with sepsis severity and mortality risk. These findings might select neonates at risk who will need more intensive monitoring and therapy (125). Thus, inflammatory markers might serve as prognostic parameter for severity of neonatal sepsis and mortality. A further study by Kurul et al. (126) on LOS showed that application of a decision tree incorporating inflammatory markers (IL-6, PCT, CRP) reached a diagnostic accuracy of nearly 88%.

Serial measurement of infection markers are thought to improve the diagnostic sensitivity of these tests. The combination of an early sensitive marker with a late specific one might enhance the diagnostic accuracy of the markers (58). Serial physical examination has been suggested as an alternative or additional tool to serial determination of inflammatory markers (127). For us this is quasi a “conditio sine qua non” in the treatment of seriously ill septic neonates. Current evidence suggests that the use of promising diagnostic markers like CD11b, CD64, IL-6, IL-8, PCT, and CRP, either alone or in combination, might be helpful when considering to discontinue antibiotics at 24–48 h of onset of the suspected infection process. In case of an infant that remains clinically well waiting for the definitive microbiological results would not be any more necessary. However, none of the current diagnostic markers are sensitive and specific enough to support the decision to withhold antibiotic treatment (58).

Even if these issues are resolved the crucial factor seems to be the difficulty to define the clinical usefulness of infection markers from the findings of the current literature (58). Mehr et al. (71) stated, back in 2000, that the heterogeneous methods of laboratory measurement and the wide variations in data analysis including cut-off values and the resulting differences in reported conclusions precluded the possibility of performing a meaningful meta-analysis. Problems that remain an issue even today (20). Reliable cut-off values are either lacking or there is an abundance of different cut-offs proposed for the same marker, both renders a potential diagnostic test wearisome to apply clinically (58). For a marker to serve as a routine diagnostic tool, high comparability and reproducibility across different laboratories is required (58).

### Future Aspects and Conclusion

There are few reports on the use of proteomic analysis from patients with sepsis, and the results have not been validated by well-established techniques (128). A recent review identified nearly 200 proteins in response to sepsis by proteomic analysis of septic blood, of whom some might serve as sepsis markers (129). The problem with proteomic analyses that identify specific proteins and peptides by random sampling of disease and control plasma from different patients and from different clinical settings is the retrospective interpretation of findings (130). For early identification of septic neonates we are faced with the same old problems of each biomarker as demonstrated in this review. In addition, this is further true for metabolomics in septic patients (131).

The clinical usefulness of pediatric heart rate in predicting clinical deterioration (e.g., pediatric sepsis) is limited by the lack of consensus among warning systems, consensus-based guidelines, and evidence-based studies as to what constitutes abnormal heart rate in the pediatric age group (132). The authors of this recent review concluded that current studies on heart rate variability do not adequately discriminate children with sepsis from those without. Maybe only in combination with biomarkers a better interpretation of the findings is possible or vice versa.

In conclusion, despite lots of promising inflammatory markers, the clinical ability to discriminate between infected and uninfected neonates remains to be a challenge, and antibiotics are often initiated in ill term and especially preterm infants. Hence, for the early diagnosis IL-6 (from cord blood or peripheral neonatal blood) and later repetitive measurements of CRP seem to be helpful in the diagnosis of neonatal sepsis considering the clinical aspect of the neonate, its gestational age, maternal risk factors, and the time of sampling.

## Author Contributions

JE and BR were responsible for the writing of the manuscript. ER for the literature search, table, and figure. BR finally edited the last version of the manuscript. All authors listed have made a substantial, direct, and intellectual contribution to the work and approved it for publication.

## Conflict of Interest

The authors declare that the research was conducted in the absence of any commercial or financial relationships that could be construed as a potential conflict of interest.

## Publisher's Note

All claims expressed in this article are solely those of the authors and do not necessarily represent those of their affiliated organizations, or those of the publisher, the editors and the reviewers. Any product that may be evaluated in this article, or claim that may be made by its manufacturer, is not guaranteed or endorsed by the publisher.
